# Optimizing laboratory X-ray diffraction contrast tomography for grain structure characterization of pure iron

**DOI:** 10.1107/S1600576720014673

**Published:** 2021-02-01

**Authors:** Adam Lindkvist, Haixing Fang, Dorte Juul Jensen, Yubin Zhang

**Affiliations:** a Department of Mechanical Engineering, Technical University of Denmark, Kongens Lyngby, 2800, Denmark

**Keywords:** iron, grain mapping, X-ray diffraction contrast tomography, laboratory X-rays

## Abstract

Laboratory diffraction contrast tomography (LabDCT) is a recently developed technique for 3D nondestructive grain mapping using a conical polychromatic beam from a laboratory-based X-ray source. The effects of experimental parameters, including accelerating voltage, exposure time and number of projections used for reconstruction, on the characterization of the 3D grain structure in an iron sample are quantified. The experiments were conducted using a commercial X-ray tomography system, ZEISS Xradia 520 Versa, equipped with a LabDCT module; and the data analysis was performed using the software package *GrainMapper3D*, which produces a 3D reconstruction from binarized 2D diffraction patterns. It is found that the exposure time directly affects the background noise level and thus the ability to distinguish weak spots of small grains from the background. With the assistance of forward simulations, it is found that spots from the first three strongest {*hkl*} families of a large grain can be seen with as few as 30–40 projections, which is sufficient for indexing the crystallographic orientation and resolving the grain shape with a reasonably high accuracy. It is also shown that the electron current is a more important factor than the accelerating voltage to be considered for optimizing the photon numbers with energies in the range of 20–60 keV. This energy range is the most important one for diffraction of common metals, *e.g.* iron and aluminium. Several suggestions for optimizing LabDCT experiments and 3D volume reconstruction are finally provided.

## Abstract 2

A parameter study of laboratory X-ray diffraction contrast tomography (LabDCT) has been performed to clarify the effects of various experimental parameters on the 3D reconstruction of the grain structure. Recommendations for optimizing LabDCT experiments are given.

## 1. Introduction   

Laboratory X-ray diffraction contrast tomography (LabDCT) is an emerging characterization technique allowing nondestructive 3D crystallographic orientation mapping (Bachmann *et al.*, 2019 ▸; King *et al.*, 2013 ▸, 2014 ▸). It adapts principles of synchrotron DCT (Ludwig *et al.*, 2008 ▸; Reischig *et al.*, 2013 ▸) and has been implemented in a commercially available X-ray microtomography (µ-CT) microscope (Holzner *et al.*, 2016 ▸; McDonald *et al.*, 2017 ▸, 2015 ▸). LabDCT has been demonstrated to be a powerful tool for studying polycrystalline materials in three and four dimensions (*x*, *y*, *z*, time) with a spatial resolution of at best 5–10 µm and an angular resolution of 0.1° (McDonald *et al.*, 2017 ▸; Oddershede *et al.*, 2019 ▸; Sun *et al.*, 2017 ▸, 2019 ▸).

**Figure 1:**
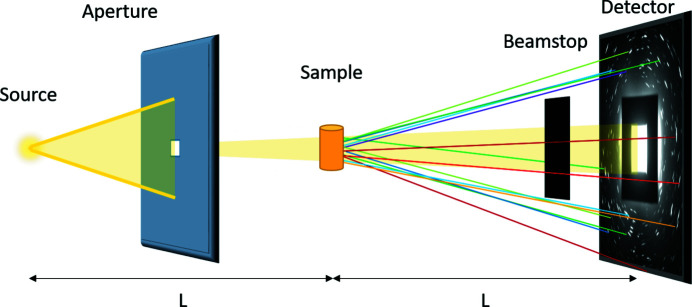
Schematic of the LabDCT setup as it is implemented on a commercial X-ray microscope, ZEISS Xradia 520 Versa.

Unlike synchrotron DCT or 3D X-ray diffraction (3DXRD) (Margulies *et al.*, 2001 ▸; Poulsen *et al.*, 2001 ▸) (also known as high-energy X-ray diffraction microscopy; Li *et al.*, 2012 ▸), where monochromatic, parallel, high-flux synchrotron X-rays are used, LabDCT utilizes polychromatic, conical, low-flux X-ray beams from laboratory X-ray tubes. A schematic of the standard LabDCT setup is shown in Fig. 1 ▸. In addition to the conventional µ-CT system, an aperture and a beamstop are introduced. The former is used to confine the direct polychromatic conical X-ray beam, while the latter is used to block the direct X-rays transmitted through the sample. Typically, the distances from the source to the sample and from the sample to the detector are the same, to fulfill the Laue focusing condition. During a LabDCT experiment, the sample is rotated 360° around the vertical axis at a selected number of intervals, through which many diffraction spots from different crystallographic lattice planes of the same grain are recorded and used for indexing of crystallographic orientations and reconstruction of the grain structure in the sample in three dimensions.

The diffraction principle for LabDCT is Laue focusing, *i.e.* X-rays with different wavelengths incoming from different angles are focused by crystal lattice planes (Guinier & Tennevin, 1949 ▸; McDonald *et al.*, 2015 ▸). This is different from standard Bragg or Laue diffraction and typically leads to elliptical diffraction spots – the short axis of the spot is a result of the focusing effect and is roughly parallel to the diffraction vector, while the long axis is due to the geometrical magnification from the conical shape of the beam and is along the direction perpendicular to the short axis (Fang *et al.*, 2020 ▸). Laue focusing requires good crystal perfection of the grains, and only small imperfections can be tolerated. Therefore, LabDCT has so far mainly been used for characterizing recrystallized grains.

With polychromatic X-rays, several lattice planes from the same grain can simultaneously fulfill the Laue focusing condition at a given rotation angle. The number of rotation intervals required for LabDCT can therefore be reduced by a factor of ∼10 compared with that required in monochromatic synchrotron DCT or 3DXRD. Furthermore, with synchrotron DCT or 3DXRD, the sample has to be rocked over a small angular range at a given rotation angle to integrate the intensity and account for any small orientation imperfections of the grains. With a continuous X-ray energy spectrum in LabDCT, diffraction intensities from grains with low crystal imperfection can easily be integrated by X-rays with slightly different energies and incoming angles at a fixed rotation angle, without sample rocking. Both of these factors contribute to reducing the data acquisition time so that LabDCT experiments can be realized with the fewer photons available from laboratory X-ray sources. However, a long exposure time of 3–5 min per projection (in contrast to 0.5–1 s for synchrotron DCT and 3DXRD) is typically used to ensure a signal-to-noise ratio good enough for resolving the shape of the diffraction spots. In order to ensure that enough diffraction spots are present to index the crystallographic orientations and resolve the grain shapes with a high resolution, typically at least 120–360 projections are used. LabDCT measurements can thus take 5–10 h or even days.

For a given experiment with a fixed total measurement time, there is a trade-off between the number of rotation steps and the exposure time per projection. The optimized condition for these two parameters is quite different for samples with different grain sizes. Also, in a LabDCT experiment, one *hkl* spot from a grain can be seen multiple times at different rotation angles, as they come from X-rays with different energies and at different angles (King *et al.*, 2013 ▸). How these spots from the same (*hkl*) plane of the same grain affect reconstruction is unknown, especially if some of these spots are redundant and can be skipped by reducing the number of projections. Additionally, the highest X-ray tube power is normally chosen to maximize the photon flux at each energy bin of a polychromatic X-ray spectrum. However, the spectrum of the polychromatic beam itself is affected also by the input accelerating voltage (Birch & Marshall, 1979 ▸). For different materials, the energy ranges of the X-rays that are most useful for the diffraction are different. For a given material with a certain average grain size, it is therefore not clear how to optimize all these experimental parameters.

The aim of the present work is to study the impacts of different experimental parameters, including accelerating voltage (maximum energy of the incoming X-rays), exposure time and number of projections, on the quality of the 3D reconstruction by conducting a series of LabDCT measurements on the same sample volume. The X-ray energies and intensities of individual diffraction spots from the same grain are determined with the assistance of a forward simulation model (Fang *et al.*, 2020 ▸), as the basis for comparing different experimental conditions. The results obtained are important for understanding the influence of each parameter on the diffraction spots from grains with different sizes, and therefore important for optimizing LabDCT measurements.

## 2. Experimental   

### 2.1. Material and LabDCT measurement   

The commercially pure iron (99.95 wt%) sample used in this study was annealed at 1123 K for 2 h, after which it was fully recrystallized with an average grain size of 75 µm and a nearly random texture. The sample shape was a cylindrical rod with a height of 30 mm and a diameter of ∼0.5 mm.

The measurements were performed using a ZEISS Xradia 520 Versa X-ray microscope equipped with a LabDCT imaging module. The sample was placed 14 mm from the source and 14 mm from the detector (see Fig. 1 ▸), utilizing the Laue focusing geometry. An aperture with a size of 375 × 375 µm and a beam stop of 2.5 × 2.5 mm were used. An absorption scan was performed in order to provide the exact size and shape of the illuminated volume. The detector used for the LabDCT measurement was a CCD camera behind a CsI scintillator of 150 µm coupled with a 4× objective lens, with an array of 2032 × 2032 pixels, providing an effective pixel size of 3.36 µm. Pixel binning was used for all LabDCT scans, effectively combining 2 × 2 pixels into one (resulting in an effective pixel size of 6.72 µm) to sacrifice resolution for a higher signal-to-noise ratio. The X-ray source was a sealed transmission X-ray tube with a tungsten anode. The source had a diameter of approximately 3–4 µm and is treated as a point source in the following analysis. Although there are peaks near the characteristic *K* lines of W in the spectrum, they are not expected to significantly affect the results since the vast majority of the X-rays used for the diffraction are generated by *Bremsstrahlung*.

**Figure 2:**
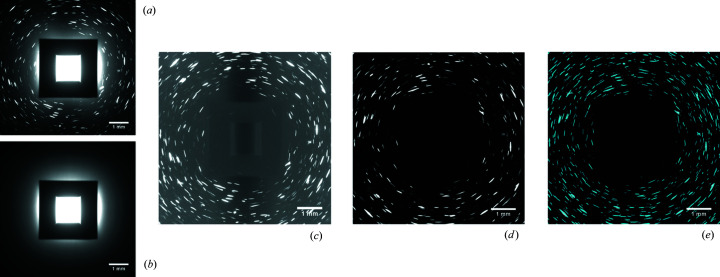
Example showing the processing steps each projection goes through in order to identify spots generated by diffraction of the sample. The data from the scan (*a*) are first divided by the data from a reference scan without the sample, known as the flat-field image (*b*), and then multiplied by 100, providing an image without the intensity resulting from the direct beam (*c*). A rolling median correction is applied to (*c*) to remove additional noise, resulting in (*d*); the spots are finally segmented by using a Laplacian of Gaussian method (*e*). The result of the aforementioned steps is a binary image where the identified spots are 1 and the background 0. The scale of each image is related to the size of the detector. Note that the sample projection most clearly visible in (*c*) has been magnified by a factor of 2.

The LabDCT scans were acquired with 121 projections collected in 3° steps with an accelerating voltage of 120 kV and exposure times ranging from 20 to 400 s. Reconstructions using every other and every third projection of the scan with an exposure time of 400 s were also performed. An additional scan was carried out with an accelerating voltage of 150 kV. The power applied to the X-ray tube was 10 W for all the scans, which is the maximum power allowed by the instrument to ensure a small size of the source. The electron current therefore changes from 83.3 µA for 120 kV to 66.7 µA for 150 kV. The experimental parameters are summarized in Table 1 ▸. A typical raw projection obtained with an exposure time of 400 s and an accelerating voltage of 120 keV is shown in Fig. 2 ▸(*a*).

For each scan, a single reference image was collected with the same instrument settings but without the sample. The projection obtained from this reference scan is commonly referred to as the flat-field image and contains some of the background intensities that come primarily from the direct beam and the inelastic interaction of the direct beam with the beamstop, but nothing from the sample [see Fig. 2 ▸(*b*)]. The LabDCT data used for reconstruction were then obtained by dividing the raw diffraction data by the flat-field image. After the division, the value of each pixel was multiplied by 100 to tune the majority of the values to a range that is more appropriate for image analysis (*i.e.* 0 to 255). The resulting image [see Fig. 2 ▸(*c*)] has a comparatively uniform background.

These normalized projections were then imported into the software *GrainMapper3D*, developed by Xnovo Technology ApS, for the spots to be segmented and subsequently reconstructed into a 3D orientation map. The normalized diffraction images were first processed with a rolling median correction (over 11 sequential images) to remove the background intensity [Fig. 2 ▸(*d*)], similarly to what has been done in synchrotron DCT (Johnson *et al.*, 2008 ▸).

The diffraction spots were then segmented using a background subtraction threshold of 4 followed by a filtering using a Laplacian of Gaussian (LoG) filter. A LoG filter works by first smoothing the image using the Gaussian kernel; this also removes the majority of the local noise from the image. The remaining large intensity gradients in the image are related to the spots. Rapid intensity changes are therefore highlighted by applying the Laplacian operator to the Gaussian smoothed image (Kong *et al.*, 2013 ▸). The diffraction spots in each region connected to these large intensity changes are then obtained by applying a threshold, which is defined as a percentage of the maximum intensity in the individual region in each LoG-processed image (Lind, 2013 ▸; Spont & Cardelino, 2015 ▸). A Gaussian standard deviation σ = 2.5 pixels and a threshold percentage of 10% were used. The spots with a size smaller than 10 pixels were finally removed. This set of parameters for segmentation was chosen by visually inspecting the segmentation results for all the data sets and tuning the parameters to a level which provided a suitable compromise between spot sensitivity and noise reduction. The final segmented binary images [see an example in Fig. 2 ▸(*e*)] were used for reconstructing the 3D volume. To minimize the effects of these predefined variables on the reconstruction results, they were kept the same for all the grain reconstructions.

The volume retrieved from the absorption contrast scan was divided into cubic voxels with a side length of 2.5 µm for the 3D reconstruction of the grain structure using a fast geometric indexing algorithm (Bachmann *et al.*, 2019 ▸). The lattice parameter of the sample was set to be 2.8665 Å. The four strongest {*hkl*} families of lattice planes were used for the indexing. Completeness values are designed to reflect the confidence index of reconstructed voxels and are defined as the fraction of the forward-projected signals observed in the measured data (Bachmann *et al.*, 2019 ▸). The minimum completeness was set as 45%, below which the indexing will be rejected, while the trust completeness was set as 85%, above which the indexing is accepted without further attempts to improve the indexing. The fast geometric indexing applies to every voxel in the whole sample volume. A parameter termed maximum level is used in *GrainMapper3D* to control the degree of sampling. A lower value of maximum level corresponds to a finer sampling for the indexing. A more detailed explanation is given by Bachmann *et al.* (2019 ▸). Maximum levels of both 1 and 2 were used, but there was no significant difference between the two, so only reconstructions using a maximum level of 2 will be reported here.

### 2.2. Forward simulations   

**Figure 3:**
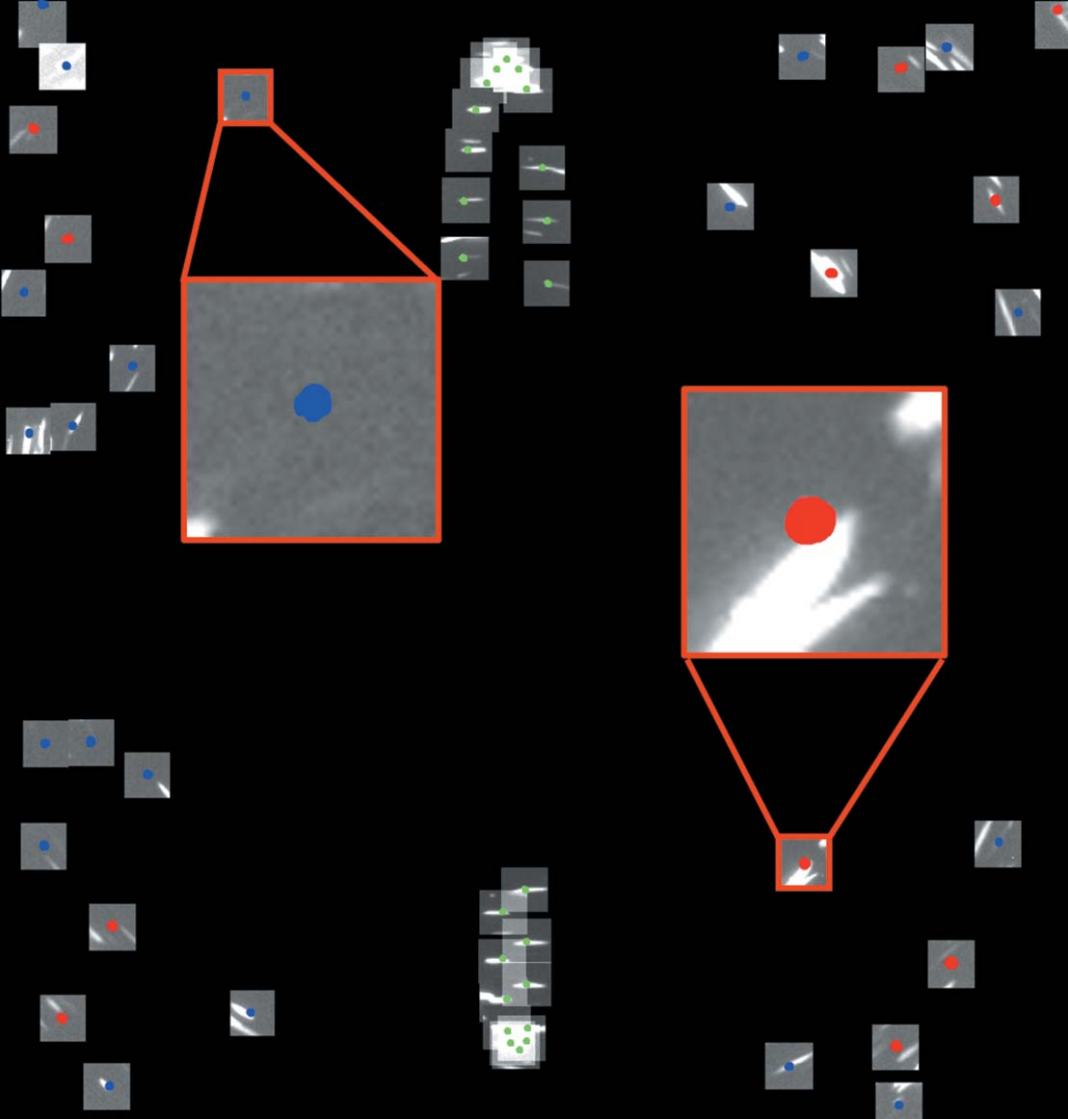
All spots used to index a small grain in data set E3 [marked by arrow in Fig. 8(*b*)] superimposed into one single image. The colored spots represent the shape computed by the forward simulation. Red corresponds to reflections from {110}, green from {200} and blue from {211}. The magnified insets show two typical cases of how the simulated spots relate to the measurements of grains considered false positives: the simulated spot matching the background (left inset) and the simulated spot matching an experimental spot from another grain, creating overlap between the two (right inset).

A polyhedron-meshing-based forward simulation model as described by Fang *et al.* (2020 ▸) was used to analyze the details about individual diffraction spots, including from which grains and which (*hkl*) planes they originate as well as the sizes, intensities and energies of the X-rays producing the spots. For the simulations, the experimentally determined 3D grain structures were used as input to calculate the X-ray energies for each diffraction spot, and to relate that to the experimentally measured intensity of the same diffraction spot. In the model, an X-ray energy spectrum from a W target was used to calculate the intensity of the diffraction spots, taking both sample absorption and detective quantum efficiency into account. More details about the analysis procedure are given by Fang *et al.* (2020 ▸). To evaluate the reconstructed results, diffraction spots from grains with sizes <25 µm were generated by the forward simulation and used to find the corresponding experimental spots. If a corresponding experimental spot was more than twice the size of the simulated one, this spot was considered to be an overlapped spot. If the completeness excluding overlapped spots was lower than the minimum trust completeness, 45%, this grain was considered as false-positively indexed (see an example in Fig. 3 ▸). In some cases, the normalized intensity as a function of X-ray energy was analyzed for the diffraction spots from the same grain, and the results for the different experimental parameters were compared.

## 3. Results   

### 3.1. Diffraction patterns   

**Figure 4:**
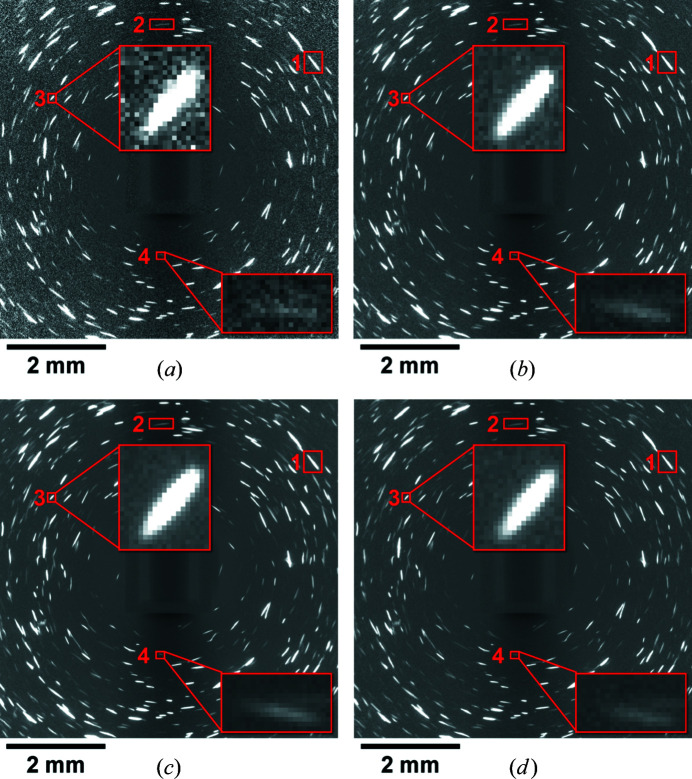
Examples of LabDCT projections collected at the same rotation angle with different experimental parameters. The images shown are the result after division by the flat-field image, similar to Fig. 2 ▸(*c*). (*a*)–(*d*) are from data sets A, C, E and D2, respectively (see Table 1). The gray scale value used for visualization is the same for all the images. The scale bars used here are derived from the pixel size, as in Fig. 2 ▸.

Examples of normalized LabDCT projections collected with the same geometrical setup and different experimental parameters are shown in Fig. 4 ▸. Here it can be seen that the diffraction image collected with an exposure time of 20 s has more of a ‘salt and pepper’ look of the background than the rest of the images, indicating a higher level of background noise. Most of the large spots are visible even in the projection with a 20 s exposure time (see an example in the upper insets in Fig. 4 ▸), while some smaller and weaker spots are more difficult to distinguish from the local background and become more visible with increasing exposure time (see the lower insets in Fig. 4 ▸).

When comparing diffraction patterns of scans using different accelerating voltages [120 versus 150 kV; see Figs. 4 ▸(*b*) and 4 ▸(*d*)], it can be seen that the spots appear slightly dimmer and smaller for the accelerating voltage of 150 kV. Upon closer inspection it is notable that this effect is more pronounced for some spots than others.

**Figure 5:**
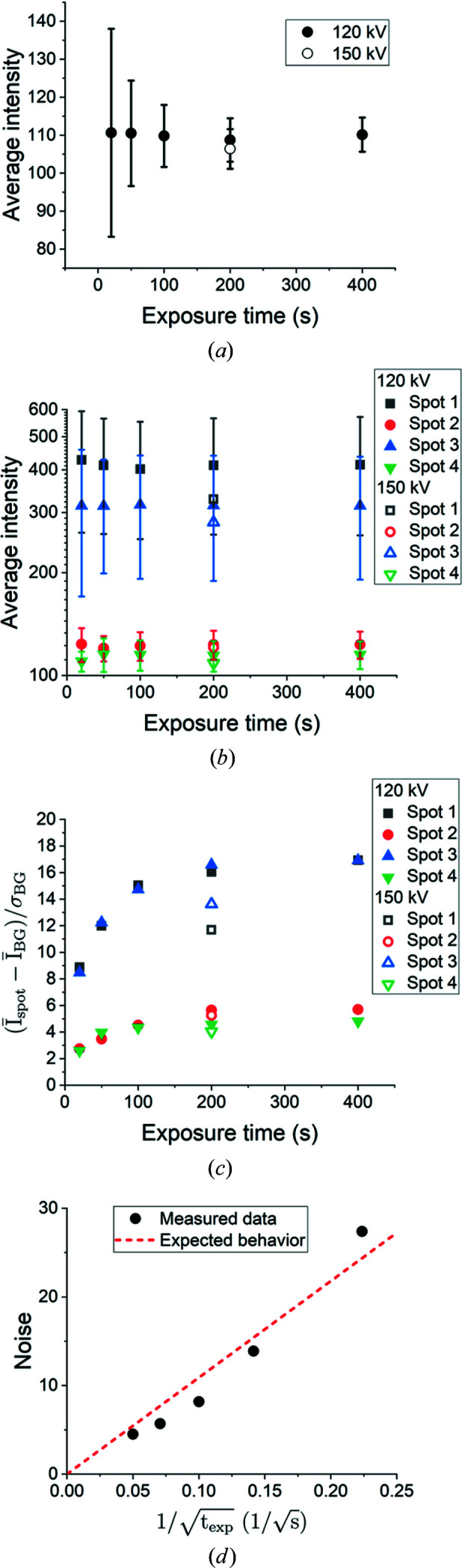
The average normalized intensity of the background (*a*) and diffraction spots (*b*), and the signal-to-noise ratio (*c*) as a function of exposure time and accelerating voltage. The error bars for the normalized background intensity indicate the standard deviation, or noise, of the background. Similarly, the error bars for the spot intensities indicate the standard deviation of the normalized intensity within the spot, which indicates the intensity range of the spot. The selected spots can be seen in Fig. 4 ▸. The noise from (*a*) is also plotted as a function of the reciprocal square root of the exposure time in (*d*). A linear relation is expected and the broken red line in (*d*) is the least-squares fitted result.

For quantification of the global average normalized intensity level and noise of the background, the normalized intensity within a total of 100 regions containing no diffraction spots across three projections was measured. The regions were at least 10 pixels from the nearest spots and ranged from approximately 1000 to 4000 pixels in size. The results of this quantification can be seen in Fig. 5 ▸(*a*). It is clear that the average value of the background is relatively constant (around 110) for all experimental parameters. However, the noise level of the background (represented by the error bars) appears to decrease as expected when the exposure time is increased. This is easily explained by Poisson counting statistics (Strum & Fenigstein, 2014 ▸), and the data [see Fig. 5 ▸(*d*)] suggest that the background noise does scale with the expected behavior:
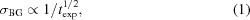
where 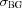 is the background noise (*i.e.* the standard deviation of the normalized global background intensity) and 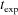 is the exposure time.

The background noise level does not appear to change significantly when the accelerating voltage is changed from 120 to 150 kV, though the average background value seems to decrease slightly as the voltage is increased [see Fig. 5 ▸(*a*)].

The average normalized intensities (calculated by dividing the integrated normalized intensity by the spot size) of the four spots marked by red boxes in Fig. 4 ▸ with different sizes are quantified in Fig. 5 ▸(*b*). It appears that the average intensities are nearly constant when the exposure time is longer than 100 s. The average intensities obtained with shorter exposure times (20 and 50 s) can be either higher or lower than those with longer exposure times.

The signal-to-noise ratio (SNR) for the spots was also calculated as
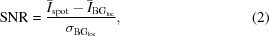
where 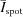 and 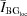 are the average normalized intensity of the spot and local background surrounding the spot, respectively, while 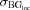 is the standard deviation of the local background. The local background is considered rather than the global background used in equation (1) since the background varies across the detector. This is most clearly seen in Fig. 2 ▸(*c*), where there is a darker central line in the background, which is likely to be a result of the inelastically scattered X-rays being attenuated by the sample. The background noise also increases with distance from the center of the detector, most clearly seen in Fig. 4 ▸(*a*). The results can be seen in Fig. 5 ▸(*c*). Here it is clearly revealed that the SNR is strongly correlated with the exposure time for each spot and that the increased voltage results in consistently lower SNR values.

Although this analysis is conducted on the LabDCT image after the division with the reference [see Fig. 2 ▸(*c*)], the results are also applicable to the noise-reduced images [*i.e.* after rolling median correction; see Fig. 2 ▸(*d*)], as in the latter case *I*_BG_ is close to zero but 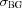 is nearly unchanged.

### 3.2. Reconstructed 3D volumes   

In total, 158–192 grains are found in the reconstructed 3D volumes based on different data sets. With longer exposure time, in general more grains are indexed, while the accelerating voltage has nearly no effect on the number of indexed grains. More surprisingly, the number of grains increases with decreasing number of projections (see Table 2 ▸).

**Figure 6:**
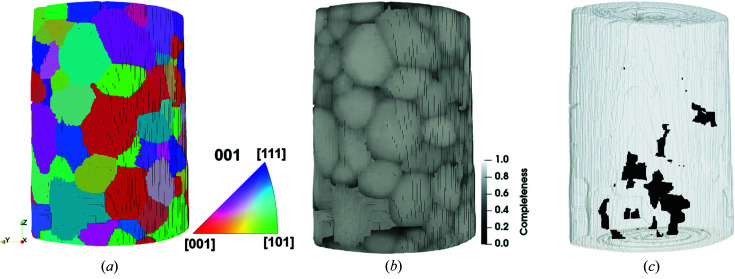
3D reconstruction of data set E1 with colors representing the grain orientation along the rotation (*Z*) axis (*a*), completeness (*b*) and the unindexed regions (*c*).

The grain reconstruction using data set E1 is shown in Fig. 6 ▸, where 171 grains with an average grain size of 74 µm are presented. The majority of the grains have completeness values close to 90%, while some smaller grains have lower values, especially those voxels close to grain boundaries [see Fig. 6 ▸(*b*)]. A small part of the volume has not been successfully indexed [see Fig. 6 ▸(*c*)]. These regions appear to be fairly randomly distributed throughout the volume. There may be several reasons for certain regions being unindexed, including the possibility that the grains in these regions are too small to generate sufficient diffraction spots.

**Figure 7:**
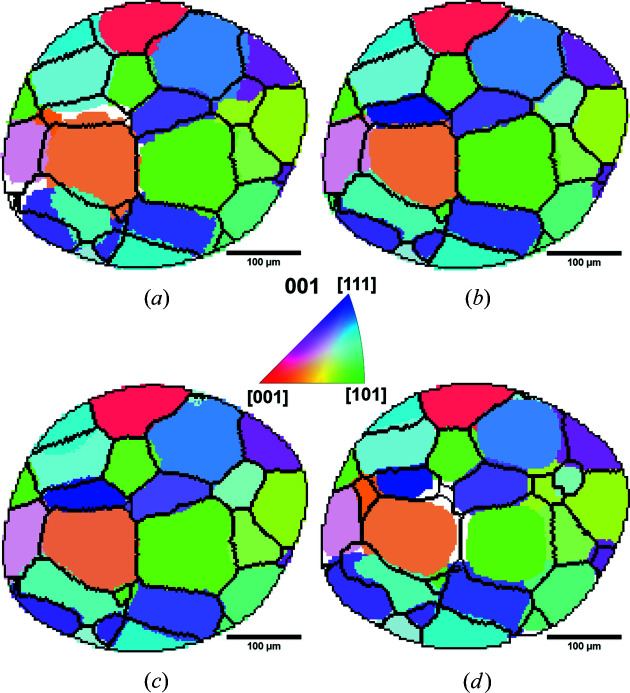
Comparisons of the same cross section of the reconstructions of different data sets. The black lines are from data set E1 in (*a*)–(*c*) and data set D1 in (*d*), while the colored grains are from (*a*) data set A, (*b*) data set C, (*c*) data set E3 and (*d*) data set D2. White areas are not indexed.

Data set E1 is expected to be the best compared with all the other data sets since the diffraction pattern used for this reconstruction has the most clearly distinguishable spots [see Fig. 4 ▸(*c*)] and the number of projections is the highest, providing more spots for the reconstruction. It is also expected that the highest number of projections will result in a better reconstruction simply because more data are available. This reconstruction is therefore used as a reference for the other reconstructions in order to compare their relative quality. An exception to this will be made when comparing the effects of the acceleration voltage using data sets D1 and D2, since these measurements were done separately from the others and are thus more easily aligned for direct comparison. Fig. 7 ▸ shows a cross section of the comparison results between the grain structures from data sets using different experimental parameters to reference data sets.

A significant difference is observed between the reconstructions using exposure times of 20 and 400 s [Fig. 7 ▸(*a*)]. The number of unindexed voxels is significantly higher for the 20 s case (see Table 2 ▸). The reconstruction is drastically improved by increasing the exposure time to 100 s, and the differences between the reconstructions using 100 and 400 s are relatively small [see Fig. 7 ▸(*b*)]. The differences are most evident in the shapes of the grains, but there are also some differences in the center-of-mass positions of some grains. The number of indexed grains increases with exposure time, especially because more small grains are reconstructed, whereas the number of false positives among the small grains appears to be constant with respect to exposure time (see Table 2 ▸). Note also that the difference in the number of small grains between data sets can be caused both by the successful indexing of the grains and by different resulting sizes of the grains; some grains with sizes close to 25 µm are counted as small grains in some data sets and not in others.

**Figure 8:**
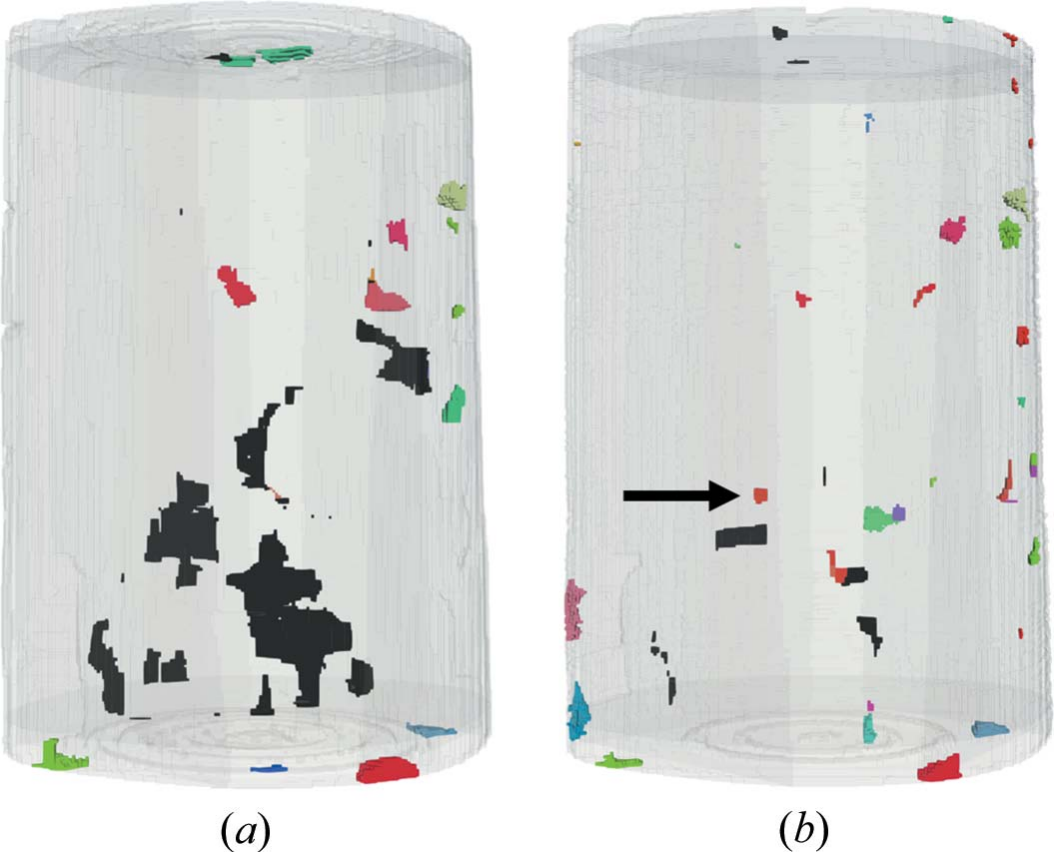
3D reconstructions of data sets E1 (*a*) and E3 (*b*), showing the grains smaller than 25 µm (colored according to orientation) as well as the internal unindexed regions (black). The black arrow in (*b*) marks a small grain, for which the diffraction spots are analyzed in detail in Fig. 3 ▸.

A similar comparison between reconstructions conducted with varying number of projections is shown in Fig. 7 ▸(*c*). It appears that the number of projections does not affect the grain structure much, except that more grains have been indexed with a reduced number of projections. All the additional grains reconstructed with fewer projections are small grains appearing at the edges of the volume (see Fig. 8 ▸ and Table 2 ▸). It can also be seen in this figure that the unindexed parts (black regions within the semi-transparent volume in Fig. 8 ▸) of the volume are smaller in the reconstructions using fewer projections. The number of false positives also increases when the number of projections decreases (see Table 2 ▸).

The grains seen in the different reconstructed volumes were paired on the basis of the orientation and position for a further quantification of the reconstruction quality. The differences in orientation and center of mass between each grain pair as well as the percentage of the shared common volume were quantified in detail and the results are listed in Table 3 ▸.

It is found that all of the 171 grains in the E1 volume are also seen in the E3 volume (including the ten false-positive grains), and the misorientation for the same grains between the two data sets is always below 0.01°, implying a very good orientation accuracy with reconstruction using 41 projections. The common volume between the volumes E1 and E3 is about 91%, indicating a reasonably good reconstruction accuracy with the 41 projections. The additional 23 grains that can be seen in the volume from the E3 data set but not in the E1 data set are all false positives. The average center-of-mass shift between the matching pairs is below one voxel (see Table 2 ▸).

The difference between the reconstructions using different accelerating voltages can be seen in Fig. 7 ▸(*d*). Data set D1 was used as a reference when comparing with data set D2 in order to directly compare the effect of changing the accelerating voltage from 120 to 150 kV without changing the exposure time. While the number of reconstructed grains is nearly unaffected by the accelerating voltage, the grain shape appears to be significantly affected. A relatively large portion of the volume is unindexed, especially around triple junctions and quadruple points.

## 4. Discussion   

### 4.1. Effects of preprocessing   

The division of each projection by its corresponding flat-field image is used in the present study, as it is suggested to be an efficient background correction procedure. This comes with the cost of losing the ‘true’ intensities of the spots, which theoretically could provide information useful for grain reconstruction as it does for synchrotron monochromatic DCT or 3D X-ray diffraction experiments. However, since the X-ray spectrum of the source and exact detective quantum efficiency of the detector are neither particularly well characterized nor stable throughout the experiment, the reconstruction algorithm used in this work does not rely on the intensities of spots. This means that the loss of information due to the flat-field correction is of no consequence for the purposes discussed here. However, there are other concerns regarding the flat-field correction, namely the potential amplification of noise in regions of particularly low intensity in the flat-field image.

**Figure 9:**
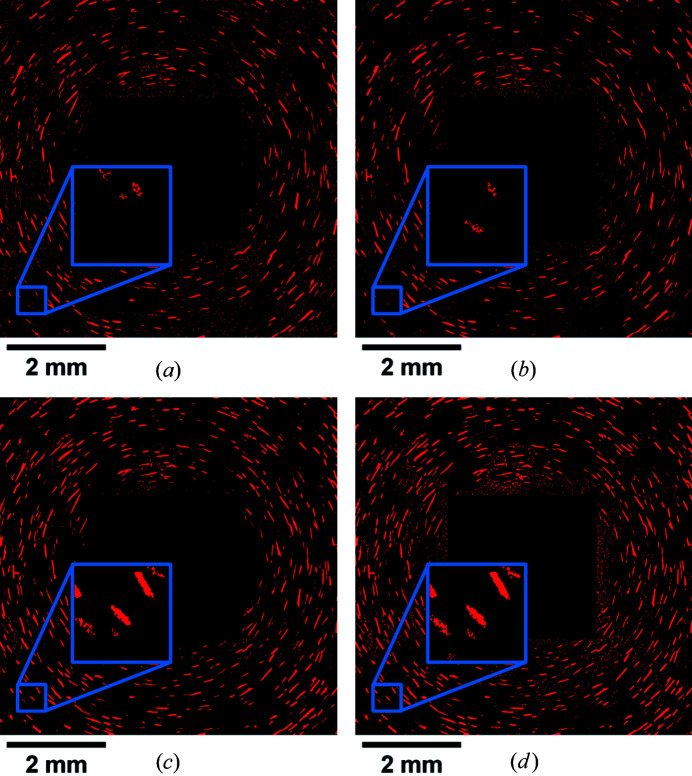
Binary images resulting from the LoG-based segmentations of data sets A (*a*), (*b*) and D1 (*c*), (*d*). The regions which have been segmented as spots are displayed in red. The images in (*a*) and (*c*) are segmented from projections normalized by division by the flat-field image, whereas (*b*) and (*d*) are not. All images have gone through a rolling median correction.

A comparison of the quality of the segmentation with and without the flat-field correction step was therefore made (see Fig. 9 ▸). While it is evident that longer exposure time leads to less noise and more segmented spots (see *e.g.* the insets in Fig. 9 ▸), it is clear that the segmentation is improved by the flat-field correction, with the notable exception of data set A. Upon closer examination of the intensity of the flat-field image used for data set A, it is found that the low exposure time resulted in some pixels having near-zero intensities. This is most pronounced near the edges of the detector, where the flat-field intensity is the lowest because these regions receive the least amount of scattering from the beamstop. This effect is also a contributing factor to the noise level of data set A, being higher than what is expected from the linear trend shown in Fig. 5 ▸(*d*). However, the noise levels close to the beamstop seem to be lowered by division of the flat-field projection even for data set A, owing to the background being bright enough to avoid near-zero values. Interestingly, this is also the region with the most inaccurately segmented pixels when the raw projections (*i.e.* without flat-field correction) are used. This is because the intensities in this region vary substantially throughout the experiment, probably because of the instability of the source. These intensity variations cannot be removed completely by the rolling median correction, resulting therefore in a large number of noisy regions in the segmented images [see *e.g.* Figs. 9 ▸(*b*) and 9 ▸(*d*)]. The reason for the clear improvement in the normalized data sets is that the magnitude of these intensity variations is significantly reduced by the division by the similarly high intensities of the flat-field image in these regions, after which the background level can be removed [see *e.g.* Figs. 9 ▸(*a*) and 9 ▸(*c*)]. The choice of whether or not to divide by the flat-field image should therefore be considered with both the exposure time and the experimental geometry in mind. It may be worthwhile to perform segmentations using both options and choose the better result for either short exposure times or geometries which will generate especially low intensities on parts of the detector, *e.g.* if the detector has a very high angular coverage. For the present study, it is evident that the division by the flat-field images has overwhelming advantages for nearly all the data sets.

The segmentation of the diffraction patterns is also a crucial part of the preprocessing. Since there is no set of segmentation parameters that will be optimal for every data set, there will always be some tuning in order to achieve a good segmentation. More specifically, the user will typically need to find a compromise between the spot sensitivity and the amount of noise being segmented as spots. The spot sensitivity in this case would reflect both the minimum size and normalized intensity of the spots that can be segmented but also the ability to segment the entirety of each spot. This means that, if different segmentation parameters are used for different data sets, it will have additional unexpected effects on the comparison of the final reconstructions. To avoid such effects and in the interest of minimizing the number of variables for the comparison between data sets, the segmentation parameters were kept constant in this study to a level that is suitable for most of the data sets. However, further tuning of these parameters as well as the development of new segmentation techniques, *e.g.* by deep learning algorithms, may further improve the accuracy of each reconstruction (Hovad *et al.*, 2020 ▸).

### 4.2. Effects of exposure time   

The quantitative analysis of the intensities of diffraction projections shows that the exposure time does not affect the average normalized intensities of the background and spots much (see Figs. 4 ▸ and 5 ▸). This is mainly because the gray values of the diffraction images are obtained by normalizing the intensities of the raw diffraction image with those of a flat-field image, the result of which is then multiplied by 100 (see Fig. 2 ▸). The intensities of both the flat-field image and the raw diffraction image scale linearly with time. This means that the resulting gray value should theoretically be 100, when there is no scattering from the sample. A slightly higher background value of 110, as seen experimentally, suggests that some inelastic X-ray scattering from the sample and/or diffraction from higher-order {*hkl*} families contributes to the background. Even though the background level (*i.e.* 110) can be removed by the rolling median correction process, the background noise will still affect the segmentation of diffraction spots.

It can also be seen in Fig. 5 ▸(*c*) that the signal-to-noise ratio increases with increasing exposure time, which is strongly correlated with the ability to properly segment spots. This is in part because some weak diffraction spots [*e.g.* the spot in the lower inset in Fig. 4 ▸(*a*)] can be blurred by the background noise. These spots may be missed after rolling median correction operation during spot segmentation, especially when the spots are small (see lower insets in Fig. 9 ▸), and are primarily responsible for the reduction in the number of reconstructed grains and the increased number of unindexed voxels [see Fig. 7 ▸(*a*) and Table 2 ▸]. However, the improved ability to resolve small spots from the background by increasing exposure time is limited, partly by diminishing returns [as seen in the signal-to-noise ratio in Fig. 5 ▸(*c*)] and time limitations. Additionally, the background noise can affect the size and outline of the diffraction spots even for the large spots (*e.g.* the spot in the upper insets in Fig. 4 ▸ where the edges of the spot with short exposure times are irregular as compared with the same spot measured with longer exposure times), which is directly related to the reconstructed shape of the grains. Similarly, the difficulty in resolving weak higher-order {*hkl*} families of the larger grains will also affect the shape information available for those grains. This effect may affect the accuracy of the shape information of large grains but is unlikely to significantly change the number of reconstructed grains.

Last but not least, the background intensity originates from the interaction between the direct beam and the sample. Even though this intensity level can be removed by post processing using, for example, a rolling median correction process, there will still be a critical level of background noise determining whether weak spots can be distinguished from the background. Smaller samples will typically lead to a lower background level due to less inelastic scattering and can therefore be beneficial for enhancing the contrast of weak spots from small grains.

### 4.3. Effects of number of projections   

The results show that reconstruction with only 41 projections gives satisfactory results. To understand this behavior, the number of diffraction spots available for the reconstruction and their corresponding *hkl*s for a typical 50 µm grain were analyzed with the assistance of forward simulations. The analysis showed that, by reducing the number of projections from 121 to 41, the number of spots with unique *hkl*s (*i.e.* removing diffraction spots from the same *hkl*) is reduced only from 42 to 41, while the total number of spots is reduced from 240 to 98. This is because, with 121 projections, one *hkl* index of a grain can result in about six spots on average during a 360° rotation. The angle range covered from the left to the right side of the detector for the present setup is ∼27°. If the diffraction vector is close to the rotation axis and a spot can be followed rotating simultaneously with the sample rotation from one side of the detector to the other, the spot can be seen in every projection and the same spot can thus be seen ten times with 3° rotation interval. For the worst case scenario, where the diffraction vector is perpendicular to the rotation axis and a spot follows a θ–2θ relation, the spot can be seen only about five times. Taking the beamstop into account, one *hkl* index of a grain resulting in on average six spots is very reasonable. The repeated diffraction spots are redundant for indexing the grain orientation but can certainly improve the reconstruction of the grain shape [see Fig. 7 ▸(*c*)]. At the same time, it is evident from the data set reconstructed using 41 projections that 1–2 spots per *hkl* index is enough for indexing the grain orientation accurately (see Table 3 ▸). If the projection number is further reduced, *e.g.* to 21, there will still be more than 40 diffraction spots per grain available for indexing the orientation (theoretically, four independent spots can determine an orientation; Chung & Ice, 1999 ▸) and reconstructing the grain shape. However, the number of unique *hkl* indices will be reduced. Both the orientation accuracy and the grain shape will be impaired and the number of false-positively indexed grains will be even higher.

The results have also shown that the reduced number of projections causes an increased amount of false-positive indexing. A similar effect is seen by examining the unindexed regions of the volume, namely that the unindexed regions are smaller when fewer projections are used (see Table 3 ▸). However, the number of correctly indexed grains in the E3 data set is still larger than all the other data sets collected with less exposure time and more projections (see Table 3 ▸). The false-positively indexed grains can be eliminated during the post processing or by optimizing the indexing routine, *e.g.* using a procedure similar to the present one involving forward simulations; the reconstruction may then be improved further. It is therefore more important to find the grains by increasing the exposure time, rather than increasing the number of projections.

### 4.4. Effects of accelerating voltage   

**Figure 10:**
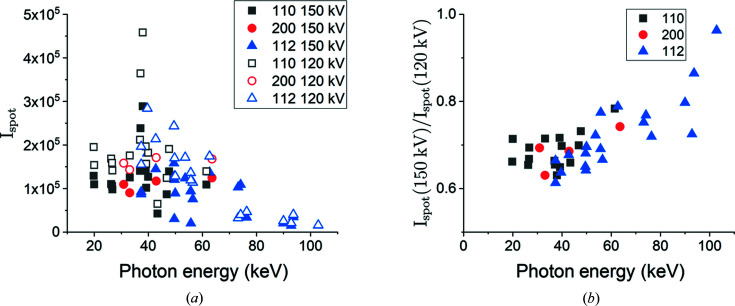
Integrated normalized intensity of spots (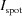) resulting from a grain with a diameter of 150 µm as a function of photon energy measured with voltages of 120 and 150 kV (*a*) and the ratio of the integrated intensities as a function of photon energy (*b*).

The accelerating voltage primarily affects the spot intensity, in contrast to the exposure time which mostly affects the background noise (see Fig. 5 ▸). Since the acceleration voltage changes the shape of the X-ray spectrum (Birch & Marshall, 1979 ▸), and not just the overall flux, it is expected that different spots are going to be affected by the changed accelerating voltage to different extents depending on the energy of the X-rays generating the spot. Forward simulations were used to analyze the spot intensities as a function of photon energy and {*hkl*} family. The results for 40 spots from the same grain with a size of 150 µm obtained with different accelerating voltages are shown in Fig. 10 ▸(*a*). Here it can be seen that the intensities of spots obtained with 150 kV are consistently lower than those with 120 kV for all energy ranges. Note that the X-ray energies for the same diffraction spot are the same at different accelerating voltages. The difference is larger at low than at high energies [see Fig. 10 ▸(*b*)]. Since the electron current at 150 kV is about 20% lower than that at 120 kV, a difference of 30–40% in the energy range 20–40 keV implies that an additional 10–20% decrease can be attributed to the change in the X-ray spectrum resulting from the increased acceleration voltage. Similarly, the difference becoming as small as 4% in the energy range of 80–100 keV implies an increase of approximately 15% in the X-ray flux resulting from the changed acceleration voltage, although the net effect of the normalized intensity is reduced due to the lower electron current.

Note also that the majority of diffraction events for the first three strongest {*hkl*} families occur in the energy range 20–60 keV. The low normalized intensity of the spots in this energy range at 150 kV is evidently an important reason for the non-indexed voxels in the reconstructed volume [see Fig. 7 ▸(*d*)].

The energy range can be estimated from the geometry of the setup along with Bragg’s law. For the present study, the sample-to-detector distance of 14 mm and beamstop width of 2.5 mm give the minimum scattering angle 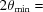 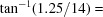 5.1° and the detector size similarly provides the maximum diffraction angle 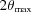 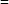 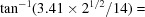 19.0°. Estimated photon energy ranges based on this angle range for various common metals and for the first three lowest {*hkl*} families can be found in Table 4 ▸. Note that all of the energy ranges are relatively large and close to one another, suggesting that the results from this study may be of use even when examining other metals.

In practice, this energy range needs to be considered together with the detective quantum efficiency and sample attenuation for optimizing LabDCT experiments. The low {*hkl*} families have high structure factors, and their corresponding photon energies are low. Since most scintillator-based detectors have the highest detective quantum efficiencies for low-energy photons while the efficiency gets significantly decreased for photon energies higher than 60–80 keV (Fang *et al.*, 2020 ▸), the energy range for the low {*hkl*} families should be prioritized by tuning the X-ray spectrum from the source, especially for samples with small grains and low atomic number. For samples with high atomic number, a comparatively higher energy range and a sample with smaller diameter is preferred.

The analysis above of the relationship between the normalized spot intensity and the energy of the X-rays producing the spot is specific to this particular combination of source and detector. The frequency of spots from different energy ranges will also depend on the geometry of the setup. However, the approach used can be applied to any LabDCT setup, possibly even to some extent before an experiment has been performed using forward simulations (Fang *et al.*, 2020 ▸), provided that the detective quantum efficiency and X-ray spectrum of the source are well characterized.

### 4.5. Optimizing a LabDCT experiment   

The results show that LabDCT experiments can be optimized with respect to the three experimental parameters studied here. The following suggestions are learned from this study:

(1) For a sample with small grains (<30 µm), a longer exposure time of *e.g.* 400 s or longer is prioritized over a large number of projections. A small sample size is preferable for reducing the sample attenuation as well as the background intensity and thereby increasing the visibility of the weak spots from the small grains.

(2) For a coarse-grained sample or for a quick overview of the larger grains (>75 µm) in the sample, a short exposure time as low as 20–50 s can be sufficient. Increasing the number of projections to more than 90–120 will only offer a limited improvement for resolving the grain shapes.

(3) For a given crystal structure, it is worthwhile to conduct a forward simulation or theoretical calculation based on a real experimental geometry to analyze the X-ray energy range for the lowest 3–4 {*hkl*} families. Such information is valuable for the choice of the optimal accelerating voltage.

(4) In general, a higher electron current is preferable compared with a higher accelerating voltage for a given maximum input power.

(5) A forward simulation on an experimentally reconstructed volume is recommended to eliminate any false-positively indexed grains.

## 5. Conclusions   

In the present study, the effects of the following experimental parameters on the quality of 3D grain reconstructions using laboratory X-ray diffraction contrast tomography have been studied for a pure iron sample: accelerating voltage (also electron current), exposure time and number of projections. It is found that, for LabDCT experiments performed using instruments similar to the ZEISS Xradia 520 Versa X-ray microscope where a W anode is used and the maximum tube power is fixed,

(i) the exposure time plays a more critical role for the 3D reconstruction than the number of projections and accelerating voltage, and should be prioritized for a given experiment;

(ii) a low number of projections such as 30–40 can be sufficient for indexing the grain orientations and resolving the grain shape with a reasonable accuracy; and

(iii) for common metals, it is recommended to maximize the electron current by limiting the accelerating voltage to a level of 100–110 kV, and thereby maximizing the photon numbers with energies in the 20–60 keV range.

While this study was limited to examining pure iron, some of the results can be generalized to other materials and the method used can be applied to any material. Last but not least, the analysis suggests that the spot overlap rate should also be considered in the reconstruction process or in the evaluation of the reconstructed result.

## Figures and Tables

**Table 1:** Experimental parameters of each data set Note that the scanning times for data sets E2 and E3 are unavailable since they were not individual scans but rather subsets of the scan resulting in data set E1. However, they can be easily estimated as half and a third of the time for E1, respectively.

Data set	Accelerating voltage (kV)	Exposure time per projection (s)	Number of projections	Total scanning time (hh:mm)
A	120	20	121	00:47
B	120	50	121	01:53
C	120	100	121	04:18
D1	120	200	121	07:20
D2	150	200	121	07:20
E1	120	400	121	13:58
E2	120	400	61	N/A
E3	120	400	41	N/A

**Table 2:** Statistics of reconstructed grains in each data set ‘Small grains’ include all grains below 25 µm, while false positives are small grains that have a completeness lower than 45% when overlapping spots are excluded.

Data set	Number of indexed grains	Number of small grains	Number of false positives	Volume fraction indexed (%)
A	158	11	11	97.9
B	164	13	9	99.0
C	168	16	10	99.3
D1	166	14	10	99.1
D2	167	15	12	96.0
E1	171	17	10	99.2
E2	181	27	19	99.7
E3	192	40	33	99.9

**Table 3:** Measurements showing the effects of changing the number of projections

Data set	Volume fraction unindexed (%)	Average misorientation from E1 (°)	Average center-of-mass shift from E1 (µm)
E1	0.81	–	–
E2	0.33	0.005	1.65
E3	0.12	0.006	2.315

**Table 4:** Estimated energy ranges for the three lowest {*hkl*} families resulting in diffraction that will hit the detector using the setup discussed here

			Energy range (keV)		
Material	Lattice parameter (Å)	Crystal structure†	1st	2nd	3rd
Fe	2.866	B.c.c.	18.5–68.7	26.2–97.2	32.1–119.0
Al	4.049	F.c.c.	16.0–59.6	18.5–68.8	26.2–97.3
Ni	3.524	F.c.c.	18.4–68.5	21.3–79.0	30.1–111.8
Cu	3.615	F.c.c.	18.0–66.7	20.8–77.1	29.3–109.0

† Body-centered cubic (b.c.c.) or face-centered cubic (f.c.c.).
